# Exploring the links between necrotizing enterocolitis and cow's milk protein allergy in preterm infants: a narrative review

**DOI:** 10.3389/fped.2023.1274146

**Published:** 2023-11-08

**Authors:** Rosemary Moak, Neal Boone, Natalie Eidson, Allison Rohrer, Mindy Engevik, Kelli Williams, Katherine Chetta

**Affiliations:** ^1^ Department of Internal Medicine, Medical University of South Carolina, Charleston, SC, United States; ^2^ Department of Pediatrics, Division of Neonatal-Perinatal Medicine, Medical University of South Carolina, Charleston, SC, United States; ^3^ Department of Pediatrics, Medical University of South Carolina, Charleston, SC, United States; ^4^ Department of Regenerative Medicine & Cell Biology, Medical University of South Carolina, Charleston, SC, United States; ^5^ Department of Microbiology & Immunology, Medical University of South Carolina, Charleston, SC, United States; ^6^ Department of Pediatrics, Division of Pediatric Pulmonology, Allergy and Immunology, Medical University of South Carolina, Charleston, SC, United States; ^7^ C.P. Darby Children’s Research Institute, Medical University of South Carolina, Shawn Jenkins Children’s Hospital, Charleston, SC, United States

**Keywords:** necrotizing enterocolitis, food allergy, inflammation, immune system, diet, formula, premature infant, neonatal intensive care unit

## Abstract

A broad range of allergic disorders and intolerance are associated with cow's milk protein in the infant diet. Allergy and intolerance to cow's milk proteins are commonly recognized in the healthy term infant, and the prevalence cow's milk protein intolerance (CMPI) varies widely but 5 challenge confirmed studies free from selection bias ranged from 1.9%-4.9%. These disorders are classified by the presence of IgE, non-IgE or T-cell-mediated signaling. Additionally, the severity of these adverse food reactions can range from mild gastrointestinal symptoms to severe sepsis-like episodes, as in the case of food protein-induced enterocolitis syndrome (FPIES). Food protein-induced intolerance in the healthy young infant lies in stark contrast to enterocolitis that typically occurs in the preterm neonate. Necrotizing enterocolitis (NEC) is a distinct progressive disease process, usually characterized by a high mortality rate, with a risk of death from 30% to 50%. While its exact etiology is unclear, its main triggers include formula (cow's milk protein), hypoxia, perfusion-related issues, and unregulated inflammation in the premature intestine. The distinction between NEC and cow's milk protein intolerance is difficult to discern in some cases. In the late preterm population, infants with colitis can have both NEC and cow's milk intolerance on the differential. In infants with multiple episodes of mild NEC, cow's milk protein intolerance may be the underlying diagnosis. In this review, we compare the pathophysiological characteristics, diagnosis and treatment of disorders of cow's milk protein intolerance with the entity of preterm NEC. This review highlights similarities in both entities and may inspire future cross-disciplinary research.

## Introduction

Bloody stools in a preterm infant are a common sign of necrotizing enterocolitis (NEC), which is the most common gastrointestinal emergency in the neonatal intensive care unit (NICU). It is the most important diagnosis to exclude in a neonate with rectal bleeding and remains a leading cause of death in the neonatal period (1). NEC has an incidence of 1–3 per 1,000 live births in the United States and is much higher in the very low birth weight population, approaching 5%–7% (2, 3). In contrast, the most common cause of bloody stool in an infant is cow's milk protein intolerance (defined in this section). It is the most common food protein-induced condition among infants and carries a more favorable prognosis, rarely leading to mortality in the infant age group (4). The prevalence of CMPI varies widely but 5 challenge confirmed studies, free from selection bias, ranged from 1.9%-4.9% (5).

The identification of cow's milk protein intolerance (CMPI; also called cow's milk protein allergy and allergic proctocolitis) is uniquely difficult in the preterm population because its presentation can often mimic necrotizing enterocolitis. Both NEC and CMPI are inflammatory conditions that are exacerbated by cow's milk protein, such as in formula, and lead to bloody diarrhea during the first months of life (6). There is an accumulation of case reports of CMPI increasing in the neonatal population including premature infants (7–9). However, there are no specific diagnostic tests that can distinguish NEC from CMPI (9). This review will explore shared developmental, immunological, and clinical factors by NEC, CMPI and variants of cow's milk protein (CMP) disease (IgE and non-IgE subgroups).

## Definitions: NEC, CMPI, and IgE-mediated milk allergy

NEC is a distinct life-threatening disease that commonly affects neonates prematurely and etiology of NEC is hypothesized to be multi factorial. Etiologic factors in NEC include genetic predisposition, intestinal immaturity, microvascular tone changes, and abnormal microbial colonization (10). Most studies suggest a major inflammatory cascade triggered by cumulative exposures to various insults like pathogenic microbes, hypoxia, microbiota dysbiosis, microvascular blood flow, can contribute to episodes of NEC (11). NEC commonly presents with abdominal distension, decreased bowel sounds, vomiting, and bloody stool. This progressive disease can be mild but may also result in surgery or death. NEC can present similarly to sepsis, but most of the time blood, urine and CSF cultures are negative. NEC can also occur in term infants, especially those with cyanotic heart lesions and cardiac disease, but for the purposes of this review, we will focus on classical presentations of NEC in the preterm infant. NEC is a clinical diagnosis and can be stratified by Bell's Modified Staging Criteria, and few laboratory markers aid in NEC diagnosis.

Food protein-induced conditions are variable in clinical presentation based on the immune response that is induced. CMPI is a non-IgE mediated food protein-induced condition that causes allergic proctocolitis. While the exact mechanism is not well understood, food antigen sensitization plays a critical role in the development of this condition. The majority of affected infants will have high levels of eosinophilic infiltrates in the gastrointestinal tissue. CMPI is a clinical diagnosis. Symptoms will be chronic and include poor feeding, irritability, bloody or mucousy stools (blood may be microscopic), loose stools, abdominal pain, poor growth, and occasionally vomiting (6, 12–14). Many of these symptoms overlap with NEC.

In contrast to CMPI, IgE-mediated cow's milk allergy is an immediate hypersensitivity reaction where symptoms generally start within minutes to 1–2 h of ingestion. IgE-mediated reactions occur after the body has been sensitized to an allergen. During this process, milk-specific IgE antibodies bind to high-affinity Fc*ε*RI receptors on both tissue mast cells and circulating basophils. During exposure to the sensitized allergen, IgE cross-linking triggers the immediate release of several cytokines and mediators, such as histamine, tryptase, and cysteinyl leukotrienes, which result in rapid symptom onset. Symptoms include urticaria, angioedema, wheezing, rhinitis, conjunctivitis, vomiting, and/or diarrhea (4, 15).

This review will specifically focus on the non-IgE mediated cow's milk protein intolerance (CMPI). This condition will be compared with preterm necrotizing enterocolitis (NEC), and we will describe the pathophysiological and clinical factors, diagnostic evaluation and treatment of both conditions, as well as highlight the challenges these diseases present in the preterm population.

## The developing preterm infant intestine

### Altered epithelium, leaky junctions, and preterm mucosa

In general, the preterm intestine is more permeable to macromolecules, has altered mucosal glycosylation, and reduced production of immunoglobulins, leading to immature innate immunity (8). Intestinal epithelial tight junction serves as the barrier to paracellular permeation of contents from the lumen to systemic circulation (16). Preterm neonates have been demonstrated to have increased intestinal permeability during the first several weeks of life (17).

The preterm intestine is characterized by high levels of baseline inflammation. Toll-like receptors (TLRs) are critical upstream gatekeepers of inflammatory activation. TLR-4 is a pattern recognition receptor (PRR), which activates the innate and adaptive immune cells. PRRs are an important component of the innate immune system as they act as first line defense of evading pathogens. Activation of TLR4 by lipopolysaccharides (LPS) from the cell walls of gram-negative bacteria or host-derived damage-associated molecular patterns (DAMPs) leads to production of proinflammatory cytokines. Several maternal conditions may affect infant TLR4 expression. In preterm labor, preeclampsia, and placental malaria, TLR4 expression is upregulated in immune cells or maternal-derived cells, which leads to aberrant production of proinflammatory cytokines at the maternal-fetal interface. High TLR-4 activity in epithelial cells results in an uncontrolled immune response and destruction of mucosal barrier by causing epithelial cell apoptosis leading to break down of the epithelial barrier integrity and translocation of the luminal organisms (18, 19). TLR4 response is important in not only circulating immune cells in the maternal systemic circulation, but also in the developing preterm intestine (18).

Alterations in the intestinal tight junction barrier is a component of the pathological cascade in the development of NEC. IL-1β and tumor necrosis α (TNF-α) are inflammatory cytokines elevated in many inflammatory diseases including NEC and can increase the permeability in the tight junction membrane (16, 20–25). Increased permeability in the tight junctions and the epithelial membrane heightens susceptibility for further inflammation, infection, and may promote antigen crossing at the intestinal membrane. Preterm infants with increased mucosal permeability could be at risk to the negative effects of excess antigen uptake across the mucosal barrier (26–28).

## Microbial dysbiosis in allergy and NEC

The intestinal microbiota is formed in the first 1,000 days of life and is sensitive to many factors such as composition of the mother's microbiota (vaginal, skin and milk), antibiotic exposures, delivery mode, and the infant's diet (29). The maternal gastrointestinal microbiota is transferred to a newborn infant at birth. However, preterm infants have a generally more dysbiotic microbiome (30). The gut microbiota in preterm infants is characterized by limited microbial diversity and delayed colonization (30–32). Preterm infants have increased *Enterococci, Staphylococci*, and *Enterobacteriaceae* (*Enterobacter, Escherichia, and Klebsiella* spp) and have less diversity of microbial constituents when compared to term infants (30, 31, 33–39). During vaginal delivery, the mother's microbiota is the main source of microorganisms colonizing newborns (29). Some studies have reported alterations in the infant microbiome due to delivery method (vaginal or c/section), but these differences were not significantly different after 2–3 months of life in the term infant (31). Diet appears to play a larger role than delivery method in the infant gut microbiome. In breastfed newborns, *Bifidobacterium* appear as early as day 2 of life and by the second week is the predominant bacterial genus in the GI tract. Infants fed formula have increased *Escherichia, Clostridia, Bacteroides,* and *Enterobacteriaceae* (30, 40–47). These predominate until solid foods are introduced and infants are weaned from breast milk. By 2–3 years of age, the infant's gut microbiota is stabilized and composition resembles adult microbiota, with predominance of *Bacteroidetes*. In summary, the natural development of gut microbiota is disturbed by many factors: delivery mode, infant formula feedings, other environmental chemicals and antibiotics which can promote dysbiosis (48).

### Allergy and the microbiome

It is still unknown how allergy is affected by the gut microbiome. Most theories suggest the gut microbiome acts on host metabolism and adaptive immunity (49). Studies in human cohorts support the influence of dysbiosis in promoting food allergy, and limited data suggest that dysbiosis occurs early in life, even preceding the onset of sensitization (50). Allergy prevalence (e.g., food allergy, atopic dermatitis, asthma) has increased in recent decades and intestinal dysbiosis is increasingly recognized as an underlying factor. In contrast to the *hygiene hypothesis* which proposes that lack of microbial exposure in early life drives allergy disease, the *microbiota hypothesis* of allergy development suggests that the gut microbiome and intestinal dysbiosis during the first few months of life affects the immature immune system, impacting health from childhood and into adulthood (29, 50). Therefore, the increase in allergy prevalence may be influenced beyond the “hygiene hypothesis” by dysregulation of intestinal microbiome causing a loss of diversity and exposures in the first few months of life (29).

Both breastfeeding and vaginal modes of delivery are protective against allergy development by favorably influencing the formation of the infant’s intestinal microbiota and shield against allergy development (29). The intestinal microbiota in these infants has an early predominance of *Bifidobacterium,* which is a species isolated from the intestines of healthy breastfed infants and human milk. It may have substantial influence on the development of immune tolerance. Interestingly, intestinal microbiota of children with allergies in comparison to healthy children shows that children with allergic disease, primarily have a decreased diversity of their gut microbiota and low amount of *Bifidobacterium, Lactobacillus,* and *Bacteroides* (29). The relationship between infant gut microbiota and allergy has been described. These allergic responses are thought to be mediated by bacterial production of short-chain fatty acids (SCFA), including butyrate, propionate, and acetate. These intermediate SCFA may be functioning to induce tolerance by acting on dendritic cells, increasing T-regulatory cells, and increase IgA production (51). Butyrate can act directly on various cells through G protein receptors, increasing tolerance responses and downregulating the production of a multitude of inflammatory cytokines (IL-1β, IL-6, IL-17) (52, 53).

Early intestinal dysbiosis may negatively affect the development of immune tolerance by interrupting the mechanisms regulating between Th1 and Th2 cells (29). Intestinal dysbiosis may upregulate pro-allergic process and increased risk of allergy (29). The Th2 division of the adaptive immune response dominates in allergic disease. The microbiota plays an important role in generating an immune phenotype that involves the maturation of Th1 response and development of T regulatory (Treg) cells which suppress the Th2 phenotype (29, 54).

Ling et al. suggest *Clostridia* species are protective in the development of food allergy. While there was no difference in in the microbial diversity, they discovered increased levels of *Clostridium sensu stricto* and *Anaerobacter* and decreased levels of *Bacteriodes* and *Clostriudium XVIII* in infants with IgE-mediated food allergy (50, 55, 56). However, Kalliomäki et al. showed that a decrease in the number of *Bifidobacterium* species and increase in *Clostridium* species observed in 3-week-old newborns was associated with development of atopy (confirmed by skin prick tests) in the first year of life. There were also distinct patterns of neonatal gut microflora in infants in whom atopy was not developing. Allergy may be a result of an under-regulation of CD4+ T regulation, and overregulation of TH2 pathways (29, 57).

### Necrotizing enterocolitis and the microbiome

Multiple cohort studies relying on 16s sequencing, have reported that there is an abundance of pathobiont species in preterm stool as compared to term infants. Before the onset of NEC, the abundances of *Clostridium sensu stricto* from *Clostridia* class were significantly higher in early-onset NEC subjects compared to controls. In late-onset NEC, *Escherichia/Shigella* among *Gammaproteobacteria* showed an increasing pattern prior to disease onset and was higher in cases than controls before NEC onset (58). Additionally, in rodent models of necrotizing enterocolitis, stool and stool microbes from infants with necrotizing enterocolitis is an essential component to recapitulate models for NEC (59). The dysbiosis of microbiome in very low birth weight infants likely increases the risk of infections and inflammatory processes in the setting of poor mucosal barrier function. In a recent study, sepsis causing pathogens were isolated from stools of 7 of 11 infants, and fecal and blood samples were monitored. The organisms identified were not normal members of normal gut microbiota (*Group B streptococcus, Serratia marcescens*, and invasive *E. coli*). Both late-onset sepsis and NEC were associated with microbiomes dominated by *Proteobacteria* and *Firmicutes*. Developmental immaturity of innate immune function may occur in VLBW infants which can result in proinflammatory cascade. Bacterial translocation across the epithelial barrier is linked with excessive TL4 signaling that produces inflammation and necrosis characteristic of the disease (60). NEC is likely a result of regulated *innate* immune responses from dysbiosis coupled to decreased mucosal protection, leading to overactive inflammatory signaling and bacterial translocation.

Both NEC and CMPI have similar patterns of gut dysbiosis: increases in gram negative species like *Proteobacteria spp*. and overall decreases in microbial diversity. Interestingly, some case reports show that food allergies occur after the diagnosis NEC. The impact of prematurity on microbial diversity last years after NICU exposure (32), which may have implications for developing allergy in the young child. The establishment of commensal microbiota influences the infant’s immune system development. Innate and acquired immune system development in infants requires interactions and controlled inflammation in the developing gut. TLR activation results in release of cytokines and immunoregulator cytokines. The enteric effects of abnormal colonization can interfere with normal processes in the VLBW population. There are potential developmental variances in intestinal epithelial responses resulting in decreased immunoregulation and increased inflammation, both which can contribute to NEC. In addition, the microbiota can alter the systemic immune system. Systemic autoimmune disease or allergies are influenced by immune balance orchestrated by the gut depending on the microbial species. If normal microbiota development is altered, particularly in VLBW infants, there can be increased risk of later enteric disease, defects in immune tolerance, atopy, and asthma. There is high quality evidence (low-moderate certainty) that supplementation of commensal microbes (probiotics) in preterm infants reduces the risk of NEC (61). Another recent study observed that blood CD4+ T-cells (naïve and active) were reduced in term infants with CMPI after supplementing with the probiotics Bifidobacteria, suggesting probiotics could regulate T cell response in this disease (61, 62). Additionally, a 2021 meta-analysis concluded probiotics may have moderate quality evidence in accelerating tolerance of CMP. However, more powerful studies are needed to determine the effective dose and treatment (63). Immunological effects of the abnormal microbiota profile that can occur in VLBW infants as they develop include disruption of TH1/TH2/TH17/Treg immune balance. This could illuminate the relationship between inadequate commensal colonization in VLBW infants and NEC or atopic disease (60).

## Milk antigens in allergy and NEC

Cow's milk is often the first foreign protein exposure in infants, likely contributing to it being the most common food allergy affecting term infants (64). Since CMPI is propelled by dietary antigens resulting in mucosal inflammation damage, this increases exposure to allergic events or may predispose a patient to NEC (64). Preterm infants, with generally more intestinal permeability, may allow for intestinal absorption of toxic luminal antigens, increasing their risk to an inflammatory disease such as NEC (65).

### Cow’s milk protein exposure and NEC

CMP has been continually implicated in the pathogenesis of NEC. Currently, formula is avoided in the NICU for these reasons. Preterm and VLBW infants require more calories, protein, and minerals beyond human milk, and these nutritional requirements are met with human milk fortifiers (64). Historically human milk fortifiers have been formula-based but now commercial human milk based human milk fortifier is available. These newer non-bovine fortifiers have been introduced with high optimism. Sullivan et al. found lower rates of NEC in extremely preterm infants who were fed exclusive human milk-based diet (including human milk-based fortifier) compared to infants fed bovine based milk fortifier or formula (66). However, these fortifiers have yet to be compared head-to-head within a high quality, blinded randomized controlled trial adequately powered for the outcome of necrotizing enterocolitis. Studies supporting the avoidance of bovine-based fortifiers have linked cow’s milk protein antigen to death and morbidity in preterm infants (67). Yet, it is unclear which specific milk protein is implicated, and if there is a difference in outcomes between intact or extensively hydrolyzed bovine proteins that are used in modern fortification strategies with formula-based extensively hydrolyzed fortifiers. However, it is well known that mother’s milk is especially protective against NEC in very preterm infants. Additionally, there is a greater risk of developing NEC when given formula compared with donor human milk when mothers own milk is not available (68).

### Allergen sensitization window in CMPI

CMP antigen sensitization likely begins earlier than previously thought. With the onset of gastrointestinal symptoms appearing within 24 h for some term infants, intrauterine sensitization is being increasingly considered (7, 69–71). The discovery of allergens in the amniotic and fetal blood has provided evidence of intrauterine allergen exposure through trans amniotic and transplacental transfer (7, 71–74). Szépfalusi et al. reported presence of various cow’s milk allergens (*α*-lactalbumin, *β*-lactoglobulin, casein, *α*-casein, *β*-casein, *k*-casein, bovine serum albumin) in concurrence with cytokine production in umbilical cord blood of 39 neonates (72). Further, Ward et al. presented a case of fetal sensitization to CMP and wheat and TNF-α production by cord blood mononuclear cells. These findings suggest that there may be an *inductive phase* of allergy development taking place during intrauterine life, and an *effector* phase that may occurs after post-natal re-encounter with the allergen, leading to the response. Therefore, the minority of previously sensitized newborns (from *in utero* exposure) may react poorly to subsequent post-natal exposure (75).

### Allergen sensitization in dietary exposure in CMPI

After birth, infants can be exposed to cow’s milk protein through dietary exposure. Antigen exposure can occur through the mother’s own milk (MOM) in women who consume dairy with direct CMP in breast milk or direct CM ingestion through formula (64). As previously mentioned, cow’s milk protein can lead to the development of IgE-mediated or non-IgE-mediated food allergy. Clinical symptoms and time course of reactions are the distinguishing factors in differentiating between CMPI and IgE-mediated food allergy.

In a randomized controlled trial, newborns at risk for atopy were randomized either to breastfeed and avoid supplementation with CM formula for 3 days after birth (with or without amino acid-based hypoallergenic elemental formula) or to breastfeed and receive CM-based supplemental formula. At 24 months of age, fewer infants in the breastfed/amino acid-based elemental formula group had sensitization to CM (measured by IgE >0.35 U/ml) compared to the breastfed/CM formula group [16.8% and 32.2%, risk ratio (RR) 0.52; CI 0.34–0.81]. A secondary outcome of this study was clinical food allergy to CM determined by oral challenges or strongly suggestive history of reaction in combination with evidence of IgE-mediated sensitization. CM allergy was present in 0.7% of infants in the breastfed/amino acid-based elemental formula group, while 6.6% of infants in the breastfed/CM group (RR 0.10, 95% CI 0.01–0.77) (76).

Overall, dietary milk antigens make up a majority of neonatal antigens encountered in the newborn period. Most preterm infants tolerate MOM and fortification well and do not have any negative gastrointestinal effects. It is generally unknown if and how a preterm infant may be reacting to allergens. Unfortunately, we are unable to predict those who will have a poor response to dairy allergens.

## Clinical symptoms of CMPI and NEC

### Clinical presentations

Both CMPI and NEC most commonly present with rectal bleeding (71). CMPI is generally thought of as a benign condition with low morbidity. In comparison, NEC is associated with 30%–50% mortality. NEC is a gastrointestinal emergency and often the first concern when neonates present with hematochezia or feeding intolerance. NEC has similar changes in disease such as elevated CRP and low platelets, which have been shown to be associated with the severity of NEC disease. Much like in NEC, the age of diagnosis in CMPI is inversely correlated with postnatal age in preterm infants as shown in Table 1. The delay of onset in CMPI in preterm infants may be due to the time required for infants to reach a postmenstrual age of 23 weeks when the immune mechanisms have matured enough to produce an adverse immune response (87). Symptoms of CMPI can be quite variable with some infants presenting with just one symptom (most commonly bloody stools, which can be microscopic or macroscopic) while others may have multiple symptoms. Symptoms can include poor growth, poor feeding, irritability, bloody or mucousy stools, loose stools, eczema-like rash, abdominal pain, and occasionally vomiting. CMPI has been reported in both term and preterm infants and generally presents within a week of CMP exposure in the diet. Symptoms can be mild, moderate, or severe (6, 12–14, 88). Differentiation of NEC vs. CMPI is difficult as the clinical presentation is largely variable and with various local and systemic findings (8).

**Table 1:** Case reports of preterm infant with a diagnosis of cow's milk allergy in the literature.

Case	GA	BW (g)	Feeds	Day of presentation	Symptom	Abd x-ray	Laboratory	Diagnoses^a^	Outcome	Diet
1 (77)	25	710	HMF DOL 20	20	AD	^b^	PE	CMA	Fortification changed to Nutramigen without modification of maternal diet.	EHF
2 (78)	26	965	PTF, MOM + HMF	17, 28, 61	AD, E, sluggish BS, lethargic, pale, bradycardia, desaturations, H	Nonspecific diffusely dilated; PI	PE; stool Leu	NEC 2A, CMA	Three episodes of enterocolitis with PI that improved with NPO and ABX. Transitioned to AAF after the third episode.	AAF
3 (79)	27	1,150	HM, CMF DOL 42	48	H, mucinous stools	Non-specific changes	PE, Colonic biopsy with EI	CMPI	Improved after NPO and casein hydrolysate-based formula.	HF
4 (77)	27	910	HMF DOL 32	41	H	Nml	PE	NEC 1, CMA	Improved after NPO and ABX. Feeds restarted with MOM fortified with AAF without change in maternal diet	MOM, AAF
5 (77)	28	1,145	HMF DOL 10	14, 30	AD, H	Mild, diffuse, small bowel distension; no PI	PE	NEC 1, CMA	Improved after NPO and ABX. Restrained MOM fortified with Nutramigen without dietary restrictions.	MOM
6 (80)	28	1,133	HM + Formula	25	H, AD	PI, dilated bowel	PE, FE, eleavted inflammatory markers, Th	NEC	Ex Lap revealed NEC, performed ileostomy	^b^
7 (81)	30	1,340	PTF/Fortified HM	8	H, mucinous stool	Nml	PE, anemia, biopsy with EI, skin prick +, RAST + at 10 months	Allergic Colitis	Made NPO. Continued to have bloody stools on EHF requiring 3 blood transfusions. Improved on AAF.	AAF
8 (82)	30	965	HM + HMF (DOL 8)	10	lethargy, E, AD, H, mucous stools	Nml	PE	Allergic Colitis	Improved with NPO, but symptoms reoccurred when restarting BM. Improved on AAF.	AAF
9 (82)	30	1,340	HM + HMF (DOL 8)	10, 27	AD, +FOBT, tenderness	Nonspecific diffusely dilated bowel loops, PI	PE	NEC 2A	Improved with NPO then symptoms reoccurred after fortification. Treated for NEC then transitioned to AAF.	AAF
10 (83)	31	^b^	CMF	30	E, H, AD	Nml	anemia, FE, elevated fecal calprotectin	CMA	Required a blood transfusion and improved with ABX and EHF	EHF
11 (83)	31	^b^	CMF		H		Fecal eos, elevated fecal calprotectin	CMA	Improved after transitioning to EHF	EHF
12 (84)	31	1,300	HM	16	AD, +FOBT	Dilated loops	PE	NEC, CMA	Improved with NPO, ABX and AAF	AAF
13 (84)	31	1,215	HM + HMF	20	AD, +FOBT	Dilated loops	PE	NEC, CMA	Improved with NPO, ABX and AAF	AAF
14 (84)	32	1,950	HM/CMF	12	Diaper rash, H, AD	Nml	Leu, elevated CRP	NEC, CMA	Improved once transitioned to AAF by DOL 6	AAF
15 (84)	32	1,600	CMF, SBF	15,24	Watery diarrhea, H, lethargy, hypothermia, AD, decreased BS	distended loops, intramural air in stomach	Leu	NEC	Improved while NPO and started SBF. CMF at 24 DOL with systemic symptoms. Improved while NPO on ABX and AAF.	EHF
16 (7)	33	2,570	CMF	1	H, acidosis, AD	Nml	Leu, PE, anemia, EI	CMA	Improved with NPO. Bloody stool persisted with reintroduction of formula feeds. Improved after abx and AAF	AAF
17 (85)	33	2,350	MOM/CMF	2	H	Nml	PE	CMA	Improved on AAF	AAF
18 (86)	33	2,090	NPO	1	H	Dilated gas filled loops	PE, Acidosis, Leu	CMA	Improved with HM and casein hydrolyzed formula	HM
19 (84)	34	1,495	HM + HMF	14	AD, +FOBT	Dilated loops	PE, elevated CRP	NEC, CMA	Improved on AAF	AAF
20 (79)	35	2,670	MOM, CMF	10	H, mucinous stool, AD, tenderness	Abnormal gas pattern, no intramural gas	PE, rectal biopsy EI, RAST^b^	CMPI	Improved after NPO	HF
21 (6)	35	2,700	MOM	35	H, sleepiness	PI	Nml	NEC	Improved after NPO and ABX	EHF
22 (84)	36	2,500	MOM	4	H, pallor	Nml	PE, elevated CRP, anemia	NEC, CMA	Improved on AAF	AAF

AAF, amino acid formula; ABX, antibiotics; AD, abdominal distention; BS, bowel sounds; BW, birth weight; CMA, cow's milk allergy; CMF, cow's milk formula; CMPI, cow's milk protein intolerance; CRP, C reactive protein; E, emesis; EHF, extensively hydrolyzed formula; EI, eosinophilic infiltrate; Ex Lap, exploratory laparotomy; FE, fecal eosinophilia; GA, gestational age; H, hematochezia; Leu, leukocytosis; MOM, mother's own milk; NEC, necrotizing enterocolitis; Nml, normal; NPO, Nil Per Os; PE, peripheral eosinophilia; PI, pneumatosis intestinalis; SBF, soy based formula; Th, thrombocytopenia.

^a^ Includes initial and final diagnosis.

^b^ Information unavailable.

### Diagnosis of NEC

NEC is usually a clinical diagnosis that relies on key radiographical findings from plain abdominal radiography and more recently, abdominal sonography (89). Concerning findings for NEC include pneumatosis, pneumoperitoneum, and/or portal venous gas (90). To date, no biomarkers have proven to be integrated into the routine surveillance of NEC (67). Since 1997, Bell’s clinical staging of NEC and now, the modified Bell criteria have been the mainstay in diagnosing and staging NEC (91, 92).

### Diagnosis of CMPI

CMPI is a clinical diagnosis based on clinical history; there are no diagnostic tests used to make the diagnosis as it is a non-IgE-mediated disease process. Skin testing and serologic milk-specific IgE testing are not indicated unless there is immediate onset of IgE-mediated symptoms (e.g., urticaria, angioedema, cough) after milk ingestion. The diagnosis of CMPI is often based on clinical response to a milk elimination diet (71, 93, 94).

Some refractory cases of CMPI require flexible sigmoidoscopy with biopsy. Histology may show inflammatory changes (e.g., focal erythema, friable appearing mucosa, multiple surface erosions with microscopic findings which can show normal architecture without crypt atrophy or branching and focal aggregates of eosinophils in all mucosal layers, particularly the lamina propria (95, 96). When flexible sigmoidoscopies are done, tissue eosinophils are usually high in biopsies. Peripheral blood eosinophilia and/or microcytic anemia have also been reported (12, 88, 97). One meta-analysis identified peripheral blood eosinophilia in 43.8% of infants with CMPI and gastrointestinal eosinophilic infiltration in 89.3% (97).

Endoscopy with biopsy is the most sensitive diagnostic tool, however it is invasive and therefore elimination diets are the more common first line approach (95). Endoscopy should be reserved for refractory cases to exclude other causes, such as very early onset inflammatory bowel disease (IBD) (82).

CMPI has a more benign clinical course compared to NEC but in severe cases can present with pneumatosis intestinalis on abdominal x-ray, similar to that seen in NEC (6). Given that CMPI can present severely in neonates, NEC must remain at the top of the differential diagnosis (often prompting appropriate infectious work up, clinical management and antibiotics) and be ruled out before a diagnosis of CMPI is made.

Interestingly, eosinophilia can also be seen in prematurity associated with inflammatory states such as infection or NEC and is considered nonspecific (77, 98). Transient eosinophilia is reported to occur in 76% of premature infants and is commonly referred to as eosinophilia of prematurity (88). Emerging areas of research include biomarkers such as inflammatory cytokines, platelet-activating factor and fecal calprotectin, which are actively under investigation in both conditions (99–102). In addition, fecal calprotectin levels are higher in non-IgE mediated CMPI compared to IgE-mediated allergy and improve once milk is eliminated. Calprotectin can increase during inflammatory states so could be considered a useful biomarker for follow up treatment and recurrence monitoring in CMPI (103, 104).

### Treatment for CMPI and NEC

Early, accurate diagnosis of NEC is key as timing to treatment leads to more favorable outcomes (105). Management of NEC involves bowel decompression followed by bowel rest, empiric antibiotics, and possibly surgery if intestinal perforation occurs (106). The initial management of CMPI in infants is influenced by the suspicion of NEC. However, after the accurate diagnoses of CMPI, strict milk elimination from the diet and close monitoring for eventual re-introduction into the diet is standard of care (64).

### Special diets

In the breastmilk-fed infant, maternal dietary milk elimination is recommended. Maternal elimination of milk can be burdensome from both a physiologic and psychologic standpoint and mothers may or may not be motivated to do so, thus elimination diets are likely to be associated with early weaning. Additionally, there is a wide range of antigen exposure in breastmilk varying from mother to mother (107). It may take several weeks after maternal dietary milk elimination for symptoms, especially bloody stool, to resolve, as it can be found in the breast milk of lactating women for 7 days. In one small recent study using nanoflow-HPLC-tandem mass spectrometry, dietary peptides were rapidly evident at 1 h, but ultimately undetectable at 6 h after CM ingestion suggesting cow’s milk-associated proteins may clear in less time than previously suspected with others citing in as little as 72 h (107, 108). Furthermore, published CMA guidelines support continued breastfeeding through the initial phase of maternal elimination without any need for a “washout” period, unless symptoms are severe (i.e., hypoproteinemia or severe anemia). Caution should be exercised when recommending milk-free diets without a diagnosis of CMPI (108, 109). Prophylactic milk-free maternal diets may increase the risk of IgE-mediated allergy later in childhood (110).

In formula-fed infants, an extensively hydrolyzed (EH) formula is recommended in infants with CMPI. Most formula-fed infants will respond to this change; however, approximately 10% of infants will need to escalate to an amino acid-based formula, especially those who present with severe enteropathy (109). Hypoallergenic formulas are not without risks. Extensively hydrolyzed and amino acid-based formulas are generally designed for term infants and have less calcium, phosphorous, and protein than preterm infant formula. Hypoallergenic formulas generally cannot fully support the nutritional needs of preterm infants, and concentrating these formulas to higher calories results in high osmolality, which is often poorly tolerated.

### Re-introduction of cow’s milk protein and prognosis of CMPI

The prognosis for CMPI is reassuring with about half of patients able to ingest milk in their diet by age 1 and >90% with resolution by age 5 (84, 111). Generally, it is recommended to introduce milk protein in the form of baked goods prior to direct (uncooked) milk protein exposure. Once an infant is able to tolerate milk protein in their diet, a fully unrestricted diet is recommended in order to maintain milk protein tolerance and reduce the risk for developing IgE mediated milk allergy and other forms of non-IgE mediated food allergy like FPIES. This is especially important as infants with CMPI are at increased risk for developing many atopic diseases, including asthma, atopic dermatitis, allergic rhinitis, and food allergy (112, 113).

## Diagnostic overlap between CMPI and necrotizing enterocolitis

As mentioned above, symptoms of CMPI can mimic the symptoms of NEC in preterm infants. There are also reports that NEC may be the sensitizing event preceding a CMPI. On the other hand, there are reports that CMPI is the sensitizing event predisposing a neonate to NEC. Ultimately, the association between NEC and CMPI is unclear. We highlight 34 cases in Tables 1, 2 that highlight the clinical overlap between NEC and CMPI. We summarize **three theories** regarding CMPI’s association with NEC.

1. Preterm CMPI is an entity that is misdiagnosed as NEC

**Table 2:** Case reports of term infants with diagnosis of cow's milk allergy in the literature.

Case	GA	BW (g)	Feeds	Day of presentation	Symptom	Abd x-ray	Laboratory	Diagnosis	Outcome	Diet
1 (1)	37	**-**	NPO	1	Bloody gastric aspirates, H	Nml	Anemia, PE, Colonic biopsy with EI	Eosinophilic Gastroenteritis	UGI with stomach edema and sigmoidoscopy with erythema. Improved on after NPO with ABX and AAF.	MOM^a^
2 (114)	38	2,958	MOM	10	Bilious E, H	Decreased intestinal gas, nml contrast study	PE, Leu	CMA	Improved while NPO. Pt was switched to SBF with resolution of symptoms.	SBF
3 (80)	38	1,980	HM	6	Bilious E, H	gasless abdomen	PE, EI, elevated CRP, Th	NEC	UGI Nml. Ex lap revealed inflammation with proximal jejunostomy; recovered with NPO and ABX.	**-**
4 (115)	39	2,900	CMF	2, 20	H, AD	PI	PE, Acidosis, Leu	NEC, CMA	Improved after NPO with ABX and resuming oral feeds on DOL 18 provoking a second episode that improved after transitioned to EHF and MOM	EHF and HM
5 (84)	**-**	2,000	CMF, SBF	4, 19, 32	diarrea, E, hypothermia, AD, H	PI	Leu, coagulopathy	NEC	Three episodes where the first improved with feeding with SBF then had a second event after restarting CMF. The third episode was accompanied with systemic signs that improved with treatment for NEC and then tolerated EHF.	EHF
6 (69)	-	**-**	MOM	4	H	Nml; barium enema with colonic spasm	PE, Rectal biopsy EI, skin prick +, RAST +	Allergic proctitis	Improved after NPO and ABX and transitioned to EHF	EHF
7 (70)	-	3,800	CMF	2	H	Paucity of bowel gas	PE, Colonic biopsy with EI	AC	Improved after NPO and ABX with blood stools for 5 days. HF restarted on DOL 6 and discharged on DOL 8	HF
8 (70)	-	3,300	CMF	2	H	Nml	Colonic biopsy with EI	AC	Improved after NPO and ABX. HF restarted on DOL 7 and discharged on DOL 8	HF
9 (70)	-	4,000	MOM/ CMF	2	H	Nml	Biopsy with IE	AC	Improved on casein hydrolysate formula and discharged.	HF
10 (93)	-	3,250	MOM/ CMF	8	Bilious E, tarry stool	Nml	PE, Leu	CMA	UGI study showed mucosal edema. Improved after NPO then given MOM with HF.	MOM + HF
11 (93)	-	2,970	CMF	5	Bloody/ mucinous stool, bilious E	Nml	PE, Rectal EI, leucocitosis	CMA	Nml UGI. Improved while NPO and restarted on MOM on DOL 11	MOM
12 (116)	-	4,150	CMF	3	Lethargy, fever, AD, E, melena, shock, anuria	PI, pneumoperitoneum	**-**	NEC	Ex lap revealed perforated colon and NEC. Treated with small bowel resection, abx, NPO and TPN	EHF

AAF, amino acid formula; ABX, antibiotics; AD, allergic colitis; AD, abdominal distention; BW, birth weight; CMA, cow's milk allergy; CMF, cow's milk formula; CRP, C reactive protein; E, emesis; EHF, extensively hydrolyzed formula; EI, eosinophilic infiltrate; H, hematochezia; HF, hydrolyzed formula; GA, gestational age; H, hematochezia; HM, human milk; Leu, Leukocytosis; NEC, necrotizing enterocolitis; NPO, Nil Per Os; Nml, normal; MOM, mother's own milk; PE, peripheral eosinophilia; PI, pneumatosis intestinalis; RAST, radioallergosorbent test; SBF, soy based formula; Th, thrombocytopenia; UGI, upper gastrointestinal series.

^a^ Maternal milk with elimination diet.

The majority of reported cases of preterm CMPI are mistaken as NEC (Table 1). A preterm infant may be first diagnosed with suspected NEC then subsequently diagnosed with CMPI with improvement of symptoms after treating NEC (6, 71, 77, 78, 82, 84). There are also reports of term and preterm infants with CMPI who first presented with systemic symptoms concerning for NEC (6, 7, 71, 78–116). Eight of the 34 (24%) infants had pneumatosis intestinalis seen on abdominal x-rays at the time of presentation (6, 78, 82, 84, 115, 116). Coviello et al. suggest that CMPI can be mistaken as NEC with two cases of CMPI in preterm twins born at 30 weeks. Both were fed exclusively human milk diets until day of life 8 when they were fed fortified human milk. Symptoms began on day of life 10 for both infants with the first twin presenting with recurrent proctocolitis and the second with a NEC-like episode. The second twin was diagnosed with stage 2A NEC based on an abdominal x-ray showing diffuse pneumatosis. Both infants had recurrence of symptoms when restarted on human milk and subsequently transitioned to amino-acid-based formula with resolution of symptoms (82). Atkas et al. presented 5 cases of suspected CMPI that were all originally considered to be NEC (71).

2. CMPI is a pre-existing condition that increases the risk of NEC

While there are many reports of CMPI mimicking NEC, there are other reports suggesting that CMPI is a predisposing event to NEC. This could explain why some infants, but not all, have severe reactions to related to the presence of cow’s milk proteins into the diet. The three essential components for the development of NEC are (1) injury to the bowel mucosa, (2) presence of bacteria and (3) availability of metabolic substrate (115, 117). One could hypothesize an increased sensitivity to an allergen could perpetuate injury to the bowel mucosa. CMPI is due to an exaggerated immunological response either by (1) toxicity, (2) an exaggerated immunological response to milk proteins, or (3) a combination of both theories (115).

Cow’s milk formula enteral feeding is associated with NEC and sepsis (118). Dietary antigen sensitization may function in promoting and/or sustaining inflammation in both conditions (118). Chuang et al. examined in the systemic and mucosal immune compartments for evidence of bovine milk antigen sensitization in infants with NEC, which could be a potential mechanism for a direct contributory role of CMP in the pathogenesis of NEC. They suggest that T helper type I/II pro-inflammatory cytokine balance plays a role in gut immunoregulation with a propensity towards Th1 polarization in most intestinal inflammatory conditionings. It is possible that CMP may play a role in the inflammatory cascade of NEC by eliciting adaptive harmful Th1/Th2 responses. This study examined T cell response to bovine milk protein antigens in babies with NEC. They compared TH1/Th2 cytokine profile in infants who develop NEC compared to normal neonates with *in vitro* stimulation. NEC infants, compared to controls, showed elevation in baseline peripheral blood monocytes (cytokine secreting cells), vigorous mitogen responses (20–120 fold increase) for IFN-Y, IL-4, and IL5 (*p* < 0.001), strong responses to BLG (IFN-Y >IL-4/IL-5, *p* < 0.001) and some small casein responses. In the lamina propria, a small but significant increase in cytokine-secreting cells was seen in NEC infants (*p* < 0.001) with IFN-Y/IL-4 predominant response. This study shows evidence of CMP sensitization as an underlying factor in some cases of NEC, primarily in the systemic compartment, with relatively minor mucosal activation. The bovine milk protein directed effector response is of both Th1 and Th2 type at the systemic level. This contrasted markedly with findings in normal neonatal controls where both peripheral blood mononuclear cells and mucosa lamina propria cells remained quiescent under identical stimulatory conditionings. Therefore, there is sufficient translational evidence to suggest cow’s milk protein may add to the inflammatory cascade in some cases of NEC, and that these cases could be identified by underlying cow’s milk protein sensitivity. There is evidence that currently supports this theory (119).

One clinical trial has investigated the impact of maternal cow’s milk protein intake on NEC occurrence. Khalesi et al. conducted a single-center randomized double-blind study to evaluate the effect of maternal diet without bovine protein on the incidence of NEC in VLBW infants. The intervention group consisted of mothers on a milk-free diet for the first 14 days of the infants’ onset of feeding and the control group had an unrestricted diet. A maternal diet devoid of bovine protein for 14 days significantly reduced the incidence of NEC (0% NEC in the intervention group vs. 10% in the control group, *p* = 0.028). This supports the idea that exposure to cow’s milk protein can be a predisposing event for NEC given that infants receiving cow’s milk formula or exposure of antigens through breast milk were more likely to develop NEC (120). Postnatally, precocious exposure to cow’s milk proteins can increase the risk of CMPI which could further increase vulnerability in the gut wall to precipitate NEC or NEC-like illness through inflammatory damage to the gastrointestinal mucosa (120).

Interestingly, *in vitro* and *in vivo* animal studies have demonstrated that intestinal permeability is regulated by multiple factors including exogenous factors, epithelial apoptosis, cytokines and immune cells (121). Immune-mediated intestinal barrier dysfunction is thought to be critical in the predisposition to and exacerbation of several autoimmune and inflammatory conditions, including IBD, food allergy, celiac disease, and diabetes (122). Stimulation of colonic epithelial cells with IL-4 or −13 induced an increase in the intestinal permeability (123–125). Additionally, Anti-CD3-induced CD4^+^ T-cell activation in mice promotes an increase in transcellular and paracellular intestinal permeability and the release of proinflammatory cytokines such as IFN*γ* and TNF*α* (121). Chuang et al. explore the cytokine response in infants exposed to bovine milk antigen and highlights many of these cytokine responses that are seen in animal models in these studies (119).

3. Necrotizing enterocolitis can lead to subsequent cow’s milk allergy

There are cases suggesting NEC as the predisposing factor prior to the onset of CMPI in neonates. Cordova et al. suggest that persistent feeding intolerance after recovery from NEC and reoccurrence of NEC-like illness may be a manifestation of CMPI in preterm infants. After the treatment of NEC and resumption of cow’s milk protein, infants continued to manifest feeding intolerance that only resolved after an EH formula or amino acid formula was initiated. This clinical course suggests that events related to severe gastrointestinal injury and onset of NEC could be involved in the pathogenesis of CMPI in preterm infants (9).

Walther et al. presented a case of NEC which developed 3 days after starting formula. During hospitalization, the infant required bowel resection. During the recovery period, the patient was changed to an amino acid-based formula with no issues. However, upon changing to cow’s milk protein formula, the patient developed severe vomiting and excessive fluid loss from the stoma, and thus resumed the amino acid-based formula. She was readmitted at 4.5 months with bowel continuity restored. Three weeks after the re-anastomosis, cow’s milk protein formula changed and patient developed acute anaphylactic shock. This patient developed IgE-mediated milk allergy after recovery from NEC. In this patient’s case, the compromised gut mucosa could be a nidus for transfer of macromolecules and allergens which could contribute to milk protein allergy development (116).

Increased beta-lactoglobulin or casein-specific IFN-Y and IL-4 responses have been detected in recovery phases from NEC as well as the inflammatory stage which suggests that NEC was a sensitizing event (118). After the full recovery of NEC, further assessment of effector and cytokine regulatory profile has shown significant decline in beta lactoglobulin, casein-specific IFN-Y, and IL-4 cells but the regulatory TGF-B1 cells were maintained. This suggests that NEC recovery and tolerance of enteral feeds is accompanied by a switch from proinflammatory cytokines (beta lactoglobulin, casein-specific cytokines) to a profile of predominately TGF-beta regulatory cytokines. There is evidence that TGF-B1 plays a role in oral tolerance (118). Failure of a switch to regulatory cytokines, could perhaps perpetuate inflammation after NEC and contribute to the onset of CMPI or any gastrointestinal injury (118).

When differentiating pathological causes of rectal bleeding in neonates, the clinician should evaluate for systemic signs to direct management and treatment. If systemic instability is coupled with vomiting, abdominal pain and rectal bleeding, then NEC is the most important diagnosis to exclude in neonates (126). Volvulus, congenital anatomical obstruction, meconium ileus, and Hirschsprung disease must also be considered (126). In the absence of systemic symptoms in a well-appearing infant, other diagnoses to consider include CMPI, IBD, ingestion of maternal blood, anorectal fissure, infectious gastroenteritis, or lymphoid nodular hyperplasia (127).

## Conclusion

We highlighted clinical overlap in the symptoms and pathophysiology of CMPI and NEC in preterm infants. Both entities are marked by proceeding gut dysbiosis and dysregulation of the adaptive immune systems involving T-cell regulation. The windows of exposure and susceptibilities may overlap in the preterm period, both *in utero* and in the NICU. Given the similar presentation of CMPI and NEC in preterm infants, it is possible CMPI may be misdiagnosed as NEC and vice versa. Yet, the appropriate diagnostic stage and treatment for NEC must take precedence given its high mortality but the etiology of symptoms at presentation must include CMPI, especially if re-introduction of cow's milk protein leads to further symptoms. We suggest that all infants at risk of NEC and developing CMPI be screened to include a maternal dietary history and family history of atopic disease while considering best practices for nutrition and caution should be taken before recommending any elimination of diets for a breastfeeding mother. While infants often outgrow CMPI, periods of dietary avoidance of milk can increase the risk of developing atopic disease, including IgE-mediated milk allergy. Preterm infants who are discharged on hydrolyzed or elemental formulas may benefit from an allergy referral if they have persistent symptoms.

Further study of the relationship between NEC and non-IgE-mediated CMPI is needed. Cohesive guidelines are needed for prevention, diagnosis, work up and long-term follow-up particularly in premature infants.
